# A link between ghrelin and major depressive disorder: a mini review

**DOI:** 10.3389/fpsyt.2024.1367523

**Published:** 2024-03-13

**Authors:** Michał Lis, Tymoteusz Miłuch, Maciej Majdowski, Tomasz Zawodny

**Affiliations:** ^1^ Department of Internal Medicine, Endocrinology and Diabetology, Czerniakowski Hospital, Warsaw, Poland; ^2^ Faculty of Medicine, Lazarski University, Warsaw, Poland; ^3^ Faculty of Medicine, Cardinal Stefan Wyszynski University, Warsaw, Poland

**Keywords:** ghrelin, metabolic hormone, depression, major depressive disorder (MDD), mood disorder

## Abstract

Ghrelin is primarily responsible for regulating energy balance, as it increases appetite. However, in recent years, its new physiological functions have been discovered—it regulates lipogenesis, plays a role in the development of insulin resistance, and even acts protectively on heart muscle. Moreover, ghrelin was associated with many psychiatric disorders, including major depressive disorder (MDD) or schizophrenia. Ghrelin levels were elevated in patients diagnosed with depression and in patients after suicide attempts. Moreover, ghrelin was connected to depression among postmenopausal women and was shown to be a predictive marker of MDD among the elderly. Ghrelin may influence mood disorders in various ways: by regulating stress response or inflammation or altering neurotransmission in the amygdala, dorsal raphe nucleus, or hippocampus, brain regions previously connected to the pathophysiology of MDD. Genetic variants of ghrelin and its receptor have also been associated with depression. Moreover, ghrelin can interfere with the antidepressant’s action and may play a role in treatment resistance. This review highlights ghrelin’s role in depression, summarizes the existing knowledge on the subject, and presents ideas for further research.

## 1 Introduction

Major depressive disorder (MDD) is among the most common diseases of affluence. In recent studies, ghrelin has been linked to some psychiatric conditions: addictive disorders, obsessive-compulsive disorder, schizophrenia, bipolar disorder, and eating disorders (1). Ghrelin’s antagonist, leptin, has also been associated with bipolar disorder or MDD in previous research.

Ghrelin is an orexigenic hormone that primarily regulates food intake, but its many new functions have been discovered in recent years. Ghrelin regulates glucose homeostasis and might play a role in insulin resistance, another condition linked to MDD (2, 3). The hormone also influences lipogenesis and white adipose tissue utilization. Moreover, it was found to protect cardiac muscle during the recovery phase after myocardial infection by reducing sympathetic tonus (4). Ghrelin indirectly enhances the expression of insulin-like growth factor 1 (IGF-1) *via* growth hormone. The action of IGF-1 on muscle tissue, combined with increased nutrient availability (due to increased appetite), causes ghrelin to induce muscle mass growth. It was also shown that ghrelin induces the proliferation of osteoblasts and increases bone mineral density (4). Moreover, ghrelin mediates inflammation and is connected to sleep-cycle regulation, reward behavior, and memory (5).

Ghrelin binds to the growth hormone secretagogue receptor (GHSR), which is expressed in various organs of the body, including the brain, pancreas, liver, skeletal muscles, and adipose tissue, which explains its pleiotropic mechanism of action (6). Ghrelin is secreted in an inactive-deacylated form, which is later transformed by ghrelin *O*-acyltransferase to its acylated, active form (7). As a “hunger hormone”, it acts in the pituitary gland and hypothalamus in which it activates orexigenic neuropeptide Y neurons, which initiate appetite (2).

## 2 Methods

Articles on ghrelin in relation to depressive symptoms were retrieved from databases Academic Search Ultimate, ERIC, Health Source: Consumer Edition, Health Source: Nursing/Academic Edition, and MEDLINE from their inception to August 2023. The keywords were as follows: “depression” OR “depressive disorder” OR “depressive symptoms” OR “major depressive disorder” AND “ghrelin”.

The following inclusion criteria were applied for studies on humans: 1) ghrelin concentration was measured in peripheral blood, 2) patients were diagnosed with or screened for MDD according to the International Classification of Diseases (ICD) or Diagnostic and Statistical Manual of Mental Disorders (DSM) criteria, and 3) all of the participants were adults. Studies were excluded in the following cases: 1) non-English articles, 2) non-original studies, and 3) co-occurrence of chronic disorders.

## 3 How can ghrelin affect mood?

### 3.1 Stress

Stress and anxiety are major components of depression symptoms. They both activate the sympathetic nervous system and hypothalamus–pituitary–adrenal axis (HPA axis) and therefore induce the “fight or flight” response. Studies on animals revealed that ghrelin level rises in stressful settings (8, 9).

In a study conducted by Huang et al., mice were treated with chronic unpredictable mild stress (CUMS) for 8 weeks. CUMS mice had higher acylated ghrelin levels in peripheral blood and significantly higher levels of pre-proghrelin mRNA in their stomachs when compared to untreated controls (p < 0.05). The same trend was observed during the histologic evaluation of their hippocampi—the expression of both GHSR and pre-proghrelin mRNA was elevated (p < 0.01) (8). Another research concluded that ghrelin itself can induce corticosterone release. Intraperitoneal injection of ghrelin into male mice (3 mg/kg) provoked greater release of corticosterone 15 minutes after the procedure when compared to controls injected with saline (160 ± 8.5 and 95.7 ± 9.8 ng/mL, respectively). However, the same trend was not observed among female mice treated with 2 mg/kg ghrelin (10).

Current research provides evidence that excessive ghrelin secretion is mediated by adrenergic signaling, as Gupta et al. have demonstrated. In their experiment, they exposed the mice to chronic social defeat stress (CSDS) for 10 days. The procedure induced depression-like behavior in the subjects. Mice were treated with atenolol [β1-adrenergic receptor (β1AR) antagonist] every day of the experiment. On day 11, the plasma acylated ghrelin was measured, and it was found to be lowered by 36.5% when compared to that of controls not treated with β-blocker. Furthermore, mice that were given atenolol spent significantly less time in the interaction zone than matched controls, indicating exaggeration of their anxiety-like behavior (11).

Other studies have shown that ghrelin signaling protects individuals from excessive stress reactions. Mice that did not secrete ghrelin or had non-functional GHSR could not adapt to stressful situations and exhibited depressive-like behavior. Mahbod et al. assessed if handling would have had an anxiolytic effect on mice secreting no ghrelin. As they observed, ghrelin knock-out mice spent less time in open arms during the elevated plus maze test in comparison to the wild-type, previously handled mice. The outcome of this study highlights the protective and adaptive role of ghrelin in stressful settings. Moreover, mice with non-functional GHSR secreted more corticosterone 30 and 60 minutes after the stressogenic stimuli (12). It suggests that ghrelin/GHSR signaling might limit the excessive HPA activation.

Gathered evidence indicates that ghrelin might link the sympathetic nervous system and HPA axis during stress response. Ghrelin secretion is mediated by β1AR signaling, and functional GHSR is needed to prevent unrestrained corticosterone release. Therefore, ghrelin could mediate the negative feedback loop of stress response. This hypothesis is further supported by the fact that treatment with β1-blocker suppressed ghrelin secretion, which resulted in an increase in anxiety-like behavior in mice.

Adrenergic stimulation and alterations in the HPA axis are known factors engaged in the pathophysiology of depression. Patients suffering from MDD have higher concentrations of circulating norepinephrine and cortisol than healthy subjects (13, 14). Moreover, treatment with antidepressants lowers both catecholamines and ghrelin levels in serum (13, 15, 16). The role of ghrelin in stress response might constitute its link to major depressive disorder.

### 3.2 Genetics

Ghrelin–receptor gene polymorphism can also contribute to the pathophysiology of depression. Nakashima et al. provided evidence that the Leu72Met variant in the ghrelin gene was more frequent among depressed patients in the Japanese population (17). Moreover, the GHSR DNA methylation profile is also associated with depression among individuals. Cordova-Palomera et al. examined the pattern of DNA methylation in 17 monozygotic twins. The study revealed that epigenetic print in the GHSR gene varied between depressed and non-depressed subjects (18). Both studies provide evidence that genetic variations in the ghrelin signaling system constitute its role in the pathophysiology of depression.

### 3.3 Monoamines

Ghrelin, as a hormone engaged in food intake, acts on orexigenic neurons in the hypothalamus. It controls the activity of the ventral tegmental area (VTA) that projects to the nucleus accumbens (NAc). These structures consist of dopaminergic neurons and constitute a reward circuit. VTA projects to the prefrontal cortex, amygdala, and hippocampus, which are referred to as brain reward regions (19). In individuals with depression, the VTA–NAc circuit functions abnormally. The activation of NAc in response to rewarding stimuli is reduced (20).

Ghrelin was shown to increase noradrenergic and serotoninergic transmission, which is the main mechanism of action of many antidepressants (1). It would explain the alleviation of depressive symptoms after the administration of ghrelin in studies on animals. Moreover, 5-HT_2C_ receptor antagonists (such as antidepressants, e.g., fluoxetine and agomelatine) decrease serum ghrelin levels in rats (21). This effect could have arisen from the anxiolytic action of the mentioned drugs, proving ghrelin as a hormone engaged in stress response. The same trend was observed in humans with MDD treated with antidepressant or electroconvulsive therapy (ECT) (15).

Intraventricular ghrelin injection increases serotonin metabolism and promotes the expression of several serotonin receptors in both the amygdala and dorsal raphe nucleus (DRN), other brain regions that were previously connected to depression (22). Ghrelin was also found to depolarize serotoninergic neurons in the DRN. Ogaya et al. found out that ghrelin binds to its postsynaptic receptor and directly induces depolarization in DRN neurons (23). Intrahippocampal infusion of ghrelin also induced long-lasting (>120 min) synaptic plasticity. It intensified the presynaptic release of neurotransmitters and changed the amplitude of action potentials (24). Escitalopram [selective serotonin reuptake inhibitor (SSRI)] also induces neuroplasticity, and this effect is now believed to be responsible for its antidepressive mechanism of action (25).

### 3.4 Glutaminergic signaling

Signaling mediated by *N*-methyl-d-aspartate receptor (NMDAR) is involved in the development of depression (26, 27). The evidence is further supported by the antidepressive effect of NMDAR antagonists, ketamine and esketamine, used to treat treatment-resistant major depression (28, 29). Studies on animals provided insight into the relationship between glutaminergic signaling and ghrelin.

Bianconi et al. showed that ghrelin administration not only improves depression and spatial memory but also has an impact on NMDAR-mediated transmission in the brain. The study involved intrahippocampal injection of ghrelin to mice that previously underwent olfactory bulbectomy (OB). OB induces depressive-like behavior, causes memory impairment, and downregulates NMDAR. The study showed that ghrelin reversed OB’s effect on both depressive behavior and NMDAR internalization (30).

On the contrary, another research showed that ghrelin administration can antagonize the antidepressant effect of ketamine, an NMDAR antagonist. Landrigan et al. exposed mice to both ketamine and ghrelin. Mice treated with ketamine showed decreased depressive-like behavior in forced swim tests; however, when ghrelin was augmented, the effect did not persist. It was suggested that ketamine’s action is dependent on ghrelin signaling, although the coadministration of GHSR antagonists did not influence the forced swim test outcomes (31). The result of this study could explain why the body mass index (BMI) correlates positively with MDD improvement after intravenous ketamine administration (ghrelin secretion is inversely proportional to BMI) (32).

### 3.5 Inflammation

Low-grade inflammation is one of the pathophysiological components of depression. Pawar et al. have demonstrated that ghrelin administration has an impact on some of the proinflammatory cytokines. In the experiment, animals were exposed to a high-fat diet and stressors inducing depressive-like behavior. Central ghrelin administration not only improved depression symptoms but also lowered tumor necrosis factor α (TNF-α), interleukin 1β (IL-1β), and IL-6 concentrations. The reduction of inflammation was also observed during histopathological evaluation as a decrease in cell infiltration and edema in brain tissue (33). The aforementioned cytokines have been associated with depression in previous research (34–36).

**Figure 1:**
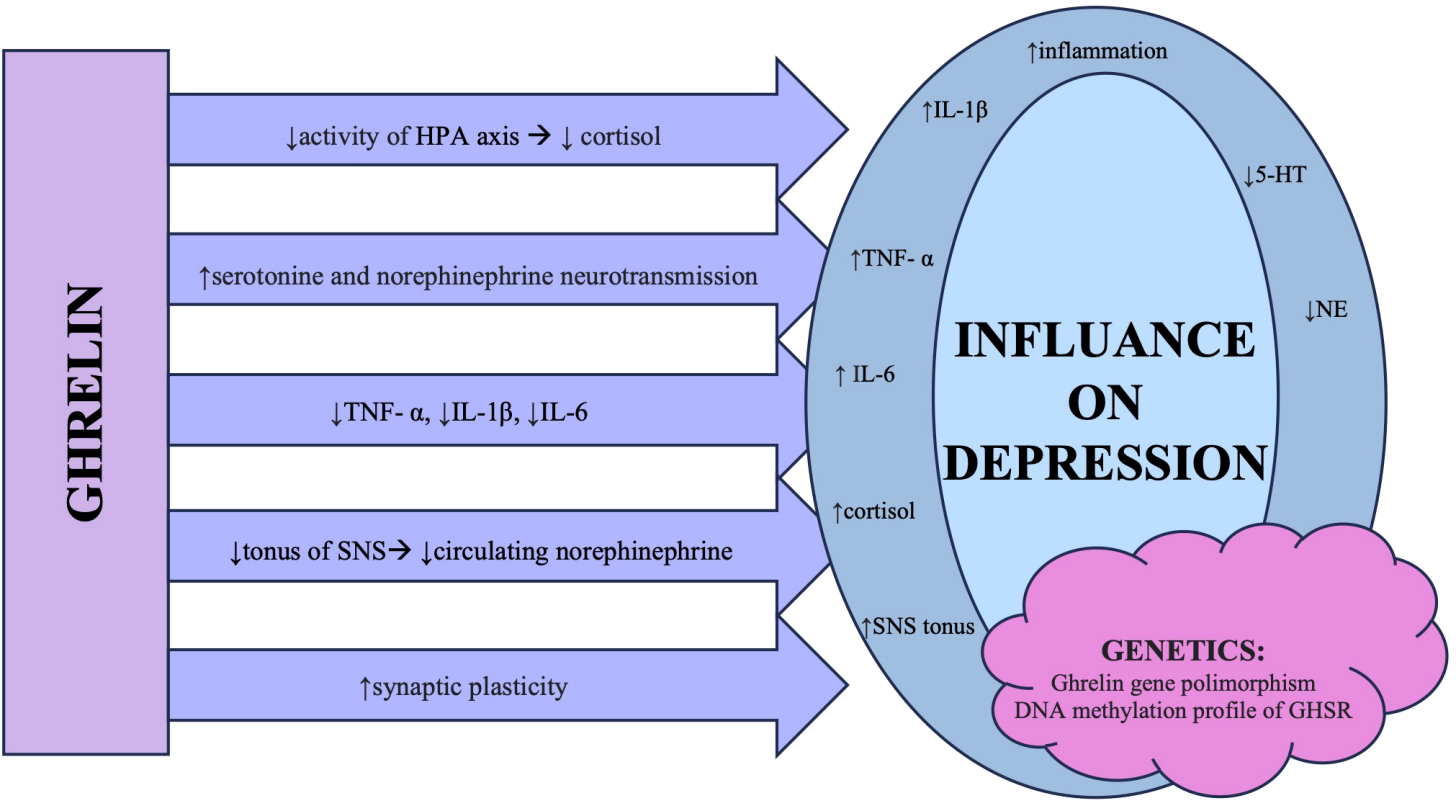
The summary of different mechanisms in which ghrelin influences changes observed in patients diagnosed with major depressive disorder. Abbreviations: HPA axis, hypothalamic–pituitary–adrenal axis; TNF-α, tumor necrosis factor α; IL, interleukin; SNS, sympathetic nervous system; 5-HT, 5-hydroxytryptamine/serotonin; NE, noradrenaline; GHSR, growth hormone secretagogue receptor.

The ways in which ghrelin influences mood are illustrated in **Figure 1**.

### 3.6 Ghrelin as an antidepressant

Several studies on animals (rats and mice) showed that ghrelin administration exhibits antidepressant effects (8, 9, 30, 31, 33, 37) expressed in outcomes of the forced swimming test, sucrose preference test, tail suspension test, or elevated plus maze test. On the contrary, some researchers declared that ghrelin induced depression-like behavior in animals (10, 38, 39). Carlini et al. reported that acute ghrelin injection alleviates symptoms of depression and anxiety, while Hansson et al. noted that its chronic administration can provoke the occurrence of depression-like behavior (37, 38). Jackson et al. performed their study on juvenile rats and observed induction of depressive symptoms 1 week after injection of ghrelin (39).

The detailed studies’ characteristics and outcomes are presented in **Table 1** and discussed in Section 5 of this review.

**Table 1:** The characteristics and outcomes of preclinical experiments regarding antidepressant effect of ghrelin’s administration.

Reference	Subjects	Dosage	Outcome
Huang 2017 (experiment 1) (8)	Male C57BL/J6 mice treated with CUMS (7–8 weeks old)	5/25/100 nmol/kg i.p. for 2 weeks	OFT: ↓ immobility (5 nmol/kg); ~ rearing numbers; EPM: ↑ entries/time %
Huang 2017 (experiment 2) (8)	Male Sprague–Dawley rats treated with CUMS (6–7 weeks old)	10 μg/rat/day, i.c.v. for 2 weeks	FST: ~ immobility; SPP: ↑ %preference; OFT: ~ rearing numbers, ~ exploration
Landrigan 2016 (31)	Male CD1 mice (8–10 weeks old)	80 μg/kg i.p. (single dose)	FST: ↓ immobility
Bianconi 2021 (30)	Female albino Swiss mice treated with OB (8.5–11.5 weeks old)	0.03/0.3/3 nmol/μL i.h. (single dose)	TST: ↓ immobility (0.3 and 3 mmol/μL); OFT: ↓ hyperactivity (0.3 nmol/μL); ORT: ↑ % exploration times, ↑ discrimination index of novelty
Lutter 2018 (9)	C57BL6/J mice	2 mg/g s.c (single dose)	FST: ↓ immobility; EPM: ↑ time in open arm
Pawar 2022 (33)	Male Sprague–Dawley rats treated with high-fat diet and diurnal rhythm disturbance	1 μg/μL intra-VTA for 5 days	FST: ↓ immobility; OFT: ↓ immobility; EPM: ↑ time in open arm, ↑ latency
Carlini 2012 (experiment 1) (37)	Female albino Swiss mice	0.03, 0.3, and 3.0 nmol/μL i.c.v. (single dose)	TST: ↓ immobility (only for 0.3 nmol/μL); OFT: ↔ crossing number
Carlini 2012 (experiment 2) (37)	Female albino Swiss mice treated with OB	0.03, 0.3, and 3.0 nmol/μL i.c.v. (single dose)	TST: ↓ immobility (only for 0.3 nmol/μL); OFT: ↓ crossing number
Hansson 2011 (38)	Male Sprague–Dawley rats	10 μg/rat/day i.c.v. for 28 days	FST: ↑ immobility, ↓ struggling, ↓ swimming; OFT: ↓ entries and time in central area, ↑ grooming
Jackson 2019 (39)	Male Sprague–Dawley rats (<2.6 weeks old)	0.2/0.5/1 nM	FST: ↑ immobility (for 0.2 or 0.5; ~ for 1 nM); TST: ↑ immobility
Jensen 2015 (experiment 1) (10)	Male NMRI mice (8–11 weeks old)	0.3/3 mg/kg i.p. (single dose)	FST: ~ immobility; TST: ~ immobility; OFT: ↑ center entries, % time in center and distance; EPM: ↑ open arm entries (only for 3.0), ↔ total number of entries to closed/open arm
Jensen 2015 (experiment 2) (10)	Female C57BL/6 mice (8–10 weeks old)	2 mg/kg s.c. (single dose)	Social home-cage: ↓ latency to reenter, ↑ visits, ↑ nose pokes

FST, forced swimming test; TST, tail suspension test; SPP, sucrose preference test; OFT, open field test; EPM, elevated plus maze test; ORT, object recognition test; i.p., intraperitoneal; i.c.v., intracerebroventricular; i.h., intrahippocampal; s.c., subcutaneous; OB, olfactory bulbectomy; VTA, ventral tegmental area.

↑, higher when compered to controls; ↓, lower when compered to controls; ~, no difference between subjects and controls.

## 4 Relationship between ghrelin concentration and major depressive disorder in humans

There are several studies on the relationship between ghrelin concentration in blood and depression among adult, unmedicated participants. Some of the authors reported higher ghrelin levels in those diagnosed with depression (15, 40, 41), while others showed decreased (42) or similar ghrelin levels (43–45) in comparison to healthy participants.

Algul et al. noted that ghrelin concentration shows a positive correlation with the severity of depression. Fasting ghrelin concentration was higher in severely depressed patients in comparison to subjects diagnosed with moderate depression or healthy controls (40).

Ozsoy et al. reported that ghrelin levels were elevated in patients with depression and normalized after treatment with antidepressants, ECT, or both. Furthermore, the method of treatment (drugs *vs.* ECT *vs.* drugs + ECT) did not have any influence on the decrease in serum ghrelin concentration (15). Kurt et al. reported the same results among depressed patients treated with ECT (46). The outcomes of the mentioned studies stay consistent with the findings of Ischitobi et al. They demonstrated that ghrelin levels are elevated in patients not responding to 8-week SSRI treatment in comparison to responders and healthy controls (16). Furthermore, Ricken et al. observed that ghrelin levels increase in non-responding patients with MDD after lithium augmentation and decrease in responding patients (47). It could suggest that ghrelin signaling mediates patients’ response to antidepressant treatment.

However, Paslakis et al. did not observe any differences in basal ghrelin concentration before and after antidepressant treatment. However, their study showed changes in ghrelin response to a standardized glucose load procedure (patients received 75 g of glucose after an overnight fasting period, and their ghrelin levels were measured at baseline and after 30, 60, 90, and 120 minutes of glucose intake). A significant difference was observed in terms of the time points of the loading procedure as well as between patients and matched healthy controls. The changes in patients’ BMI and the drug they have taken (mirtazapine *vs.* venlafaxine) showed no statistically significant influence on ghrelin concentration (48).

Active ghrelin has also been associated with postpartum depression. Baker et al. measured ghrelin levels in the 24-hour urinary samples during pregnancy and 6 weeks after labor. Women screened positive for postpartum depression 12 weeks after childbirth had higher acylated ghrelin concentrations during pregnancy. Moreover, ghrelin levels changed differently in women who were depressed during pregnancy in comparison to non-depressed subjects. The mean change of ghrelin concentration decrease from pregnancy to postpartum was less expressed among women who were depressed during pregnancy. However, the results were not statistically significant when ghrelin levels were adjusted to creatinine. It suggests that renal function should be taken into consideration while planning further research in which ghrelin is measured from urine samples (49).

There is also evidence that ghrelin levels are associated with depression severity among post-menopausal women. Naufel et al. observed that both total and acylated ghrelin concentrations were higher among those with severe depression in comparison to mildly depressed individuals. Both active and total ghrelin levels correlated positively with scores on Beck’s Depression Inventory and Patient Health Questionnaire (50).

Wittekind et al. analyzed data from LIFE-Adult-Study and included 1,092 participants whose total ghrelin levels were measured, and Center for Epidemiologic Studies Depression Scale (CES-D) scores were available. They did not observe any significant difference in regard to ghrelin concentration between depressed and non-depressed subjects. Also, there was no association between CES-D scores and total ghrelin levels (51). It should be noted that the diagnosis of depression was not confirmed by a psychiatrist nor any standardized clinical interview. There are also no data on subjects’ history of using psychotropic drugs or psychiatric evaluations. Mentioned factors could have had an influence on study results, as antidepressant treatment tends to lower ghrelin levels (15, 16, 46).

On the contrary, higher ghrelin levels were associated with a higher prevalence of depression among Japanese women. Akter et al. examined 497 participants (287 men and 210 women). They analyzed the subjects using two different cut-off points for depression in the CES-D scale (≥16 points validated for the general population and ≥19 points validated for the Japanese population). In either setting, the results stayed consistent: there was no association between ghrelin concentration in male subjects, and there was a relationship between higher ghrelin levels and higher odds for the development of depression among women (52).

In a population of older Dutch adults (55 years and older), there was no correlation between total ghrelin concentration and depressive symptoms (assessed with the CES-D scale) at the beginning of the study. However, participants with higher ghrelin levels at baseline had higher odds for depression after 3 years at follow-up, expressed specifically in participants younger than 69.7 years and with a waist–hip ratio below 0.96 (53).

Atescelik et al. conducted a study on patients admitted to the emergency department with recent suicide attempts. They reported that both acylated and unacylated ghrelin levels correlated positively with Beck’s Depression Inventory scores. Both active and non-active ghrelin concentrations were significantly higher in patients than in healthy subjects. Some of the patients had a history of psychiatric conditions (major depressive disorder, schizophrenia, schizo-affective disorder, and personality disorder); however, it did not influence the results of hormone assays between patients with different conditions and patients with no psychiatric history (54).

## 5 Discussion

In recent studies on both animals and humans, ghrelin has been associated with major depressive disorder. This link is expressed in ghrelin’s action involved in stress response and inflammation. The hormone also acts in reward regions of the brain as well as in the amygdala and DRN, regions previously connected to the pathophysiology of MDD. Ghrelin also exhibits some of the antidepressant’s mechanism of action—it influences serotoninergic and glutaminergic transmission and induces synaptic plasticity. Variations in both ghrelin and its receptor genes have also been observed in individuals with depression.

Preclinical studies on animals have revealed that ghrelin influences the results of tests assessing depression-like behavior (**Table 2**). Researchers have applied many different approaches to evaluate the hypothesis of ghrelin’s antidepressive properties, and therefore, the results are heterogenic and have their limitations. However, it should be noted that in every study in which an animal depression model was induced (by CUMS protocol, disturbed diurnal rhythm, or olfactory bulbectomy), the results stayed consistent, and ghrelin was found as an antidepressive agent independently of the dosage (route of administration, acute *vs.* chronic). Interestingly, Lutter et al. in the second set of experiments (not included in **Table 2**) did not use exogenous ghrelin but enhanced its endogenic secretion by calorie restriction, and the antidepressive effect persisted. In terms of future research, it would be crucial to induce an animal model of depression in the treated subjects. The promising results from the mentioned preclinical studies should also endorse research on humans.

**Table 2:** The summary of laboratory methods used to measure ghrelin levels in studies on unmedicated humans with healthy control group.

Reference	Biological material	Method	Temperature of storage (°C)	Ghrelin concentration in patients with MDD compared to controls
Ozsoy 2014 (15)	Serum	RAI	−70	↑
Algul 2018 (40)	Plasma	ELISA	−80	↑
Okasha 2022 (41)	Serum	ELISA	−20	↑
Chen 2021 (43)	Serum	RAI	−80	~
Giménez-Palop 2012 (44)	Plasma	ELISA (for acylated ghrelin), RAI (for total ghrelin)	−80	~
Huang 2021 (45)	Serum	RAI	−80	~
Barim 2009 (42)	Plasma + HCl	RAI	−20	**↓**

HCl, hydrochloric acid; ELISA, enzyme-linked immunosorbent assay; RAI, radioimmunoassay; MDD, major depressive disorder.

↑, higher when compered to controls; ↓, lower when compered to controls; ~, no difference between subjects and controls.

As ghrelin signaling was shown to interfere with the HPA axis, this connection should also be further evaluated. It may be a key to understanding a mechanism in which ghrelin expresses its anxiolytic mechanism of action observed in animals. There is also a need for future research on ghrelin’s influence on inflammation, as immune response is another factor that contributes to the pathophysiology of MDD, and the data available are limited.

Research on human subjects revealed that antidepressant treatment lowers ghrelin secretion. Moreover, higher levels of circulating ghrelin correlated with the development of depression in the future. Levels of these hormones were also elevated in individuals after suicide attempts and correlated positively with MDD severity among post-menopausal women. Ghrelin was also linked to postpartum depression.

The results of current studies on humans are heterogenic, and therefore, it is difficult to determine their final meaning. The problem might arise from different laboratory methods applied to measure ghrelin levels in participants (**Table 2**). Ghrelin is characterized by its short half-time, and as a relatively small protein circulating in low concentrations, it is vulnerable to pre-laboratory errors. Hence, the observed dispersion of the results may arise from different laboratory approaches: biological material used (plasma *vs.* serum), temperature of material storage (−20°C, −70°C, or −80°C), and method applied [enzyme-linked immunosorbent assay (ELISA) or radioimmunoassay (RAI)]. A consistent laboratory approach should be incorporated in future research. Moreover, it would be beneficial if both acylated and deacylated ghrelin were evaluated. This kind of systematic approach would help determine if ghrelin could be a biomarker of depression.

Those results, even if from small sample studies, show that there is a connection between ghrelin and MDD, and it might have clinical consequences. Esketamine is an antidepressant that is recommended for patients with treatment-resistant depression. In studies on animals, ketamine’s action was antagonized by ghrelin administration. Studies on humans revealed that patients with higher BMI, and therefore lower concentration of circulating ghrelin, had a higher chance for remission while being treated with esketamine. On the contrary, among patients treated with escitalopram and venlafaxine, the trend was reversed—those with higher BMI had the lowest remission rate (55). In-depth research on ghrelin signaling and antidepressant treatment could provide insight into treatment resistance mechanism, which still remains a challenge in managing treatment-resistant patients. Moreover, it could determine which antidepressant would be most suitable for a patient in a given metabolic state.

Considering that 20% to 60% of patients do not respond to first-line MDD treatment, broadening the knowledge of biological factors contributing to resistance could shorten the time to recovery. Gathered data suggest that metabolic hormones, including ghrelin, may play a role in treatment response. This possible connection is supported by the fact that ghrelin levels dropped among responders and stayed unchanged among non-responders, as Ischitobi et al. and Ricken et al. have demonstrated. Conducting research that would identify antidepressant medications with a higher response rate in a given metabolic state would benefit both patients and clinicians.

An in-depth understanding of the pathophysiology of depression is highly needed, as it could address some of the problems currently faced by practitioners and patients, which include managing treatment-resistant patients, tolerance to a drug, or weight gain following antidepressant treatment. Future research on the relationship between ghrelin and depression should address the following questions:

- Does ghrelin signaling interfere with antidepressant treatment, and if yes, could it potentially explain some aspects of treatment resistance?
- Could ghrelin concentration determine which antidepressant medication would benefit the patient the most in a given metabolic state?
- Does ghrelin exhibit an antidepressant effect in humans as it was shown in research on animals?
- Does ghrelin concentration differ between depressed and non-depressed patients, and if yes, could ghrelin be a biomarker of MDD?

## Author contributions

ML: Conceptualization, Supervision, Validation, Writing – original draft, Writing – review & editing. TM: Investigation, Writing – original draft, Writing – review & editing. MM: Investigation, Writing – review & editing. TZ: Writing – review & editing.
